# Unveiling spatial disparities in basic medical and health services: insights from China’s provincial analysis

**DOI:** 10.1186/s12913-024-10798-3

**Published:** 2024-03-12

**Authors:** Dainan Hou, Xin Wang

**Affiliations:** 1 School of Business, Minnan Normal University, Zhangzhou, China; 2 College of Life Science, Longyan University, Longyan, China; 3 School of Public Policy and Management, Tsinghua University, Beijing, China; 4 Chinese International College, Dhurakij Pundit University, Bangkok, Thailand

**Keywords:** Basic medical and health service levels, Evaluation, Spatial heterogeneity, Convergence analysis, China

## Abstract

Based on the panel data of 31 provinces (municipalities and autonomous regions) in China from 2012 to 2019, this paper constructs the evaluation index system of basic medical and health services in China from seven dimensions: medical and health facilities, health expenditure, medical services, traditional Chinese medicine hospital services, maternal and child health care, people’s health and medical security, disease control and public health. The entropy method was used to measure the level of basic medical and health services in China, and its spatial differences and convergence characteristics were further investigated. In this study, we employ the entropy weight method, σ convergence, and β convergence as our primary methodologies. The entropy weight method is used to evaluate the variability of each indicator, determine the weights of indicators, and quantify the information content of the data. σ convergence illustrates the process by which the variance of a sample decreases over time. β convergence refers to the gradual approach of variables within an economic system towards their long-term equilibrium level over time. The results show that: (1) The scores of basic medical and health services in China’s four major regions (including Northeast, East, Central and West) remain in a relatively stable state, with small fluctuations and great room for improvement; (2) There are significant regional differences in the level of basic medical and health services in China, and the intra-regional differences are much greater than the inter-regional differences; (3) There is no significant *σ* convergence observed in China and its four major regions; however, there is a notable presence of *β* convergence.

**Supplementary Information:**

The online version contains supplementary material available at 10.1186/s12913-024-10798-3.

## Introduction

This paper aims to establish a more scientifically comprehensive system of evaluative indicators to measure the level of basic medical and healthcare services in Chinese provinces from 2012 to 2019, and to analyze the spatial diversity and convergence of basic medical and healthcare service levels across China. In today’s world, health has become a crucial aspect to assess the economic and social development as well as the well-being of individuals within a region or country. As one of the developing countries, China places significant emphasis on safeguarding the health of its population. In 2016, China designated prioritizing health as one of the principles in its national overall strategic deployment. The report of the 19th National Congress in 2017 positioned a Healthy China as a national strategy. The 14th Five-Year Plan, proposed in 2020, advocates for ‘comprehensively advancing the construction of a Healthy China, placing the safeguarding of people’s health in a strategic position of priority development [1]. Given the rapid development of the social economy, there is an increasing awareness of health-related matters, and the provision of medical and health services stands as a vital determinant of overall health outcomes. The 2016 global disease burden study published by Lancet revealed that optimizing the quality of health services could potentially prevent approximately half of the 19.3 million deaths recorded in low- and middle-income countries [2]. Hence, enhancing the level of basic medical and health services while optimizing the allocation of medical resources emerges as crucial measures for elevating the global public health status.

Since the 1980s, China has embarked on a series of reforms of its healthcare system. After more than 40 years of development, the Chinese government’s medical reforms have led to rapid advancements in medical and health services, accompanied by continuous improvements in the overall standards of medical care. However, these advancements have also brought to light several challenges. One prominent issue is the difficulty and high cost associated with accessing healthcare services, resulting in an influx of patients at large general hospitals [3]. Additionally, there exists an imbalance in the distribution of medical and health resources across various regions and between urban and rural areas. This imbalance becomes particularly evident during major public health crises such as the COVID-19 pandemic [4–5].

In light of these concerns, this study aims to measure the level of basic medical and health services in China, while examining regional disparities and convergence. The findings of this research hold both theoretical and practical significance for future healthcare reforms in China, with the ultimate goal of reducing disparities in basic medical and health services across different regions and achieving equitable provision of healthcare services. Focusing on the critical issue of the level or quality of medical and health services, previous studies have predominantly addressed the following three aspects.

The first aspect involves studying the demand for or satisfaction with medical and health services from the perspective of patients. Xiao et al. [6] found elderly individuals with delayed disabilities had the highest medical needs. Jaklič et al. [7] investigated the satisfaction of patients in Gorenjska with emergency medical services (EMS), confirming the influence of the effectiveness of the EMS organizational model on patient satisfaction. Fu et al. [8] found that China’s medical resources exhibit a concentration in economically developed regions, while underdeveloped regions tend to seek medical treatment in provinces with medical resource centers. Therefore, provinces with developed medical resources will face increasing pressure in terms of medical demand.

The second aspect involves studying the supply level of medical and health services and discussing the fairness or equalization of medical services. Shashar et al. [9] found that over time, the average difference between doctors is three times that of individual practitioners. Li and Liu [10] found that the current development of rural public health services in China is unbalanced and insufficient in certain areas and regions. The development level of rural public health services across different regions exhibits a distribution pattern of “medium high,” “east middle,” and “west low,” with a spatial cluster effect. Lin et al. [11] identified significant differences in the medical and health service efficiency among 13 cities in Jiangsu Province, with a certain degree of spatial correlation.

The third aspect involves studying the reform and quality improvement of medical and health services. Chimbindi et al. [12] investigated factors related to access and utilization of health services, including access to HIV testing, among tuberculosis patients in a primary healthcare project in Hlabisa sub-district, KwaZulu-Natal, South Africa. They recommended appropriate management of HIV-TB co-infected patients. Xie [13] proposed encouraging governments at all levels to increase the investment and output of basic public services and promote a balanced and convenient utilization of these services between urban and rural areas, different regions, and diverse populations. Cao et al. [14] suggested that enhancing the efficiency of urban basic public health services should focus on the structure of health resources, particularly by increasing the number of grassroots medical and health institutions and public health personnel.

From the aforementioned three aspects of research progress, the study of medical and health service levels holds substantial theoretical and practical significance. Being a populous and rapidly developing nation, China accounts for approximately one-sixth of the global population. The Chinese government has consistently prioritized medical and health services. Since the initiation of reforms and opening up, the health status of Chinese residents has witnessed continuous enhancements, and the accessibility of fundamental medical and health services has notably improved. A diverse and multi-tiered national medical insurance system has been established, along with the formulation of a distinctive development pathway for medical and health services in China. Thus, this paper employs provincial panel data from 2012 to 2019 to assess the level of fundamental medical and health services in China utilizing the entropy method, while further examining its spatial disparities and convergence tendencies. Compared with previous research literature, the contribution of this paper is mainly reflected in three aspects:

1. Evaluation index system: This paper contributes to the establishment of a more comprehensive and robust evaluation index system for measuring the level of basic medical and health services. The constructed index system takes into account the unique context of China, resulting in a more comprehensive assessment. Notably, this study incorporates traditional Chinese medicine (TCM) hospital services into the evaluation index system, which has been rarely considered in previous similar studies. Recognizing the long-standing history and significant role of TCM in daily health management and disease treatment, it becomes imperative to include TCM hospital services within the evaluation index system.
2. Research on Regional Disparities. From a regional perspective and utilizing the method of the Theil index, this paper investigates the level of basic medical and healthcare services across the four major regions of China and their disparities, with a particular emphasis on the differences within and between these regions. In defining these regions, we adopt an approach that diverges from the conventional tripartite division of “East-Central-West” and instead analyze China as consisting of four major regions: the Northeast, East, Central, and West. The rationale for independently examining the Northeast region is twofold. Firstly, the “Methods for Dividing the Eastern, Central, Western, and Northeastern Regions” published on the official website of the National Bureau of Statistics recognize the Northeast as a distinct administrative entity. Secondly, the Northeast region demonstrates significant differences from the Eastern region in terms of geographical location, administrative boundaries, economic development level, and the production and living habits of its residents. Therefore, treating the Northeast as a separate region for study is not only instrumental in portraying a more accurate picture of the healthcare service levels across China’s various regions, but also adheres to the actual administrative and geographical distribution features.
3. The Time Span of Research Focus. This study is anchored in the new epoch of socialism with Chinese characteristics, specifically the development of China’s basic medical and healthcare services since the 18th National Congress of the Communist Party of China in 2012. Within this timeframe, China has made significant strides in both the overall scale and per capita standards of its healthcare services. The aim of this paper is to explore whether there has been a corresponding enhancement in the level of basic medical and healthcare services against this developmental backdrop, a question that has also stimulated our profound research interest. Since the 18th National Congress, China’s healthcare system has undergone transformative development, becoming one of the largest of its kind in the world, with a marked improvement in the health level of its people. Therefore, analyzing the changes in the level of basic medical and healthcare services in China from 2012 to 2019 holds not only theoretical significance but also vital practical reference for the guidance of healthcare reforms in China.
  The remainder of this paper is organized as follows: Sect. 2 provides a comprehensive overview of the existing literature, focusing on three main aspects: the conceptualization of basic medical and health services, the advancements and quality of medical and health services, and the factors influencing these services. In Sect. 3, we present our approach to constructing the index system and provide detailed explanations of our research methodology and data sources. Section 4 employs the entropy method to measure the level of basic medical and health services in China, while the Theil index is utilized to analyze regional differences and test for convergence. Finally, in Sect. 5, we present our research findings, draw conclusions, and propose strategic recommendations.

## Literature review

### Research on the connotation of basic medical and health services

Regarding the interpretation and analysis of medical and health services, various countries and regions may employ different names and definitions. Medical and health services are commonly referred to as basic health services, primary health care, basic medical and health services, and so on. The concept of basic health services was originally proposed and defined by Winslow in 1952 [15]. This concept was subsequently adopted by the World Health Organization (WHO) and has been widely employed since then. Primary health care was introduced in the Almaty Declaration in 1978 [16]. It encompasses various relevant sectors and aspects of national and community development, particularly public health, agriculture, animal husbandry, food, industry, education, housing, public works, communications, and other sectors [17]. Basic medical and health services were put forth by the World Bank in the World Development Report - Investing in Health in 1993 [17]. In this report, a new concept, namely basic public health services and clinical services, was introduced, referring to a package of fundamental preventive and medical services.

In China academic and industry research on the definition and scope of medical and health services has evolved from initial ambiguity to a clearer understanding, yet a consensus has not been reached [18]. Until 2019, the definition of basic medical and health services was officially outlined in the Law of the People’s Republic of China on Basic Medical and Health Care and Health Promotion, which was adopted at the 15th Meeting of the Standing Committee of the 13th National People’s Congress on December 28, 2019. According to this law, basic medical and health services encompass disease prevention, diagnosis, treatment, nursing, and rehabilitation services that are essential for maintaining human health, adapting to the level of economic and social development, and can be equitably accessed by citizens. These services should be provided with appropriate drugs, technologies, and equipment [19]. For the purpose of this paper, the definition stated in the aforementioned law will be employed.

### Research on the progress and service level of basic medical and health services

Scholars evaluate basic medical and health services from different regions, target groups, and supply of healthcare services. Soboka et al. [20] utilized cross-sectional data from public and private eye care institutions in Gurage district, Ethiopia, to evaluate the extent to which eye health care services in the region have achieved the objectives of the VISION 2020 Global Declaration. Schmidt et al. [21] investigated the access to healthcare for adolescents (aged 15–25 years) with chronic conditions. Their study focused on assessing the availability of healthcare services for this population. Mondaca [22] explored the fairness of health system finance and health care utilization, and examined alternative measurement methods of Chile’s access to health care. The recent research encompasses primary healthcare services across various time periods in countries including Spain, China, Iran, Thailand, India, Nigeria, and Poland [23–31]. From traditional Chinese medicine to emergency medical care in Poland during the pandemic, these studies offer an in-depth exploration of the unique challenges and solutions in medical systems around the world [23–31]. This provides a rich perspective for understanding the dynamics of global healthcare services.

### Literature review on the construction of medical and health service evaluation indicator systems and evaluation methods

In the domain of constructing evaluation indicator systems for medical and health service levels, Pan et al. [32] proposed a comprehensive system covering three key aspects: health manpower, service resources, and medical services. Zhang et al. [33] focused on three dimensions—manpower, materials, and financial resources—to build a relatively concentrated evaluation framework. Further, Shen et al. [34] expanded the dimensions of evaluation indicators, incorporating not only traditional factors such as manpower and financial resources but also the level of health management, proposing an evaluation system comprised of five dimensions: resource allocation, institutional operation, medical services, health management, and staff development.

In the selection of evaluation methods, scholars have adopted a variety of research techniques to align with the characteristics of different indicator systems and data attributes, including Analytic Hierarchy Process (AHP), Technique for Order of Preference by Similarity to Ideal Solution (TOPSIS), Relative Satisfaction Ratio (RSR), Entropy Method, Principal Component Analysis (PCA), and others. Concurrently, Data Envelopment Analysis (DEA) has also been utilized in evaluation efforts [35–36]. In addition to comprehensive evaluation methods, some studies have also employed two-period panel data difference methods. For instance, Wang et al. [37] utilized the DID method to investigate the impact of comprehensive pilot reforms in urban public hospitals on the capacity of urban medical and health services.

### Research review

To summarize, previous studies have made significant theoretical and empirical advancements, providing valuable references and inspiration for this study. However, there are certain aspects that require attention in the research on basic medical and health services levels. The following points should be considered:

1. The concept of basic medical and health services should be approached as a dynamic concept, allowing for variations in its interpretation based on the economic and cultural levels of different countries and regions. Hence, countries and regions with varying economic and cultural contexts should adapt and expand the concept based on guiding and standardized concepts.
2. In measuring the level of basic medical and health services, some studies have relied solely on cross-sectional data for evaluation, potentially compromising the accuracy of assessments. Furthermore, the establishment of an evaluation index system, encompassing indicators at all levels and appropriate evaluation methods, directly impacts the accuracy of evaluations. In this study, we employ China’s provincial panel data to enhance the sample size and extend the study’s time range. Additionally, when constructing the indicator system, we include traditional Chinese medicine hospital services in the evaluation indicator system, based on the synergy theory and China’s national conditions. In the construction of a medical and health service evaluation indicator system, this study is grounded in synergy theory and fully considers China’s specific national conditions. The paper draws on the conceptual frameworks found in existing literature and builds a comprehensive evaluation system that encompasses multiple dimensions, including facilities, cost input, service quality, maternal and child health care, the health status of the population, and disease prevention and control. Particularly, in recognition of the unique position and role of traditional Chinese medicine (TCM) services in China’s healthcare system, this paper innovatively incorporates TCM services into the evaluation indicator system, aiming to provide a more comprehensive and contextually appropriate set of indicators for China.
3. Regarding the empirical methods employed, this study draws upon certain research methods from existing literature and incorporates the Thiel index to analyze intra-regional and inter-regional differences. Furthermore, China is divided into four major regions: Northeast, East, Central, and West. It is worth noting that in some existing literature, the provinces of Heilongjiang, Jilin, and Liaoning in Northeast China were included in the East and Central regions for research purposes [30, 38]. However, considering both geographical distance and economic regions, such a regional division is deemed unreasonable. Therefore, this paper separately classifies the aforementioned three provinces as part of the Northeast region.

## Research design

### Construction of evaluation index system

It is a common research methodology to evaluate target problems by constructing an evaluation index system. Several scholars have proposed different approaches to constructing the evaluation index system for medical and health service levels, considering aspects such as input and output [39–40], input, output, and result [24], or medical and health resources, medical and health services, and health care capacity [41]. Therefore, following this established method, the present study also utilizes this approach to measure the level of basic medical and health services.

During the process of constructing a medical and health service evaluation indicator system, it is crucial to precisely define the basic medical and health service system of China and apply a composite system synergy model for research. To provide high-quality medical and health services, it is essential to have good hardware and software conditions, while the health status of the population and the degree of medical security are direct reflections of the output of medical and health service activities. Therefore, these factors must be included as key parts of the system in constructing the evaluation system.

The construction of medical and health facilities and the investment in health funds constitute two fundamental subsystems of the level of medical and health services. They represent the hardware conditions and are the combination of service entities and regional spatial health demands. These hardware conditions are the premise for the development of medical and health service software. Consequently, the evaluation system must include indicators such as the number of medical institutions, doctors, beds, health fund investments, and government health expenditures.

Medical services, traditional Chinese medicine (TCM) hospital services, and maternal and child health care constitute the software part of the medical and health service system. They not only provide intrinsic support for the basic medical and health service system but an efficient software system can also promote further development of the hardware conditions. The key indicators covered by these three subsystems include the number of patient visits, the average daily workload of doctors, the number of prenatal and postnatal examinations, and others.

As the hardware and software construction of the basic medical and health service level continues to improve, the health status of the people and the level of medical security also continue to enhance, and disease control and public health services are continuously optimized, effectively promoting the synergistic development of the medical and health service system. Accordingly, the evaluation indicators for these aspects mainly include the birth rate, mortality rate, incidence of infectious diseases, and others.

In summary, the basic medical and health service system represents a vast and intricate professional system, characterized by closely interconnected components. Hence, it is imperative to adopt a comprehensive and complex perspective when constructing an indicator system for assessing the level of basic medical and health services.

Drawing upon synergetics theory, this study takes into account the actual development of China’s basic medical and health services, in accordance with relevant laws and regulations, and references prior research in order to establish a comprehensive, reasonable, and systematic evaluation index system. The establishment and selection of the indicator system thoroughly consider the principles of synergy, operability, reality, comparability, and objectivity. Finally, an evaluation index system of basic medical and health service levels was established, consisting of seven dimensions and thirty-seven indicators. These dimensions include medical and health facilities, health expenditure, medical services, traditional Chinese medicine hospital services, maternal and child health care, people’s health and medical security, and disease control and public health (see Table 1).

**Table 1:** Evaluation index system of China’s basic medical and health service level

One-level indicators	Two-level indicators
Medical and health facilities (A)	Average number of hospitals per 10,000 people (A1)
	Average number of primary medical and health institutions per 10,000 people (A2)
	Average number of professional public health institutions per 10,000 people (A3)
	Average number of health technicians per 1,000 people (A4)
	Average number of general practitioners per 10,000 people (A5)
	Average number of beds in medical and health institutions per 1000 people (A6)
Health expenditure (B)	Proportion of total health expenditure in GDP (%) (B1)
	Total health cost per capita (CNY) (B2)
	Government health expenditure (CNY) (B3)
	Proportion of medical and health care expenditure of urban residents in consumption expenditure (%) (B4)
	Proportion of rural residents’ health care expenditure in consumption expenditure (%) (B5)
	Per capita medical expenses of outpatients (CNY) (B6)
	Per capita medical expenses of inpatients (CNY) (B7)
Medical service (C)	Average number of visits by residents (C1)
	Annual hospitalization rate of residents (%) (C2)
	Utilization rate of hospital beds (%) (C3)
	Average length of stay (C4)
	Daily Visits Per Doctor (C5)
	Daily Inpatients Per Doctor (C6)
Traditional Chinese medicine hospital services (D)	Average number of traditional Chinese medicine institutions per 10,000 people (D1)
	Average number of beds in traditional Chinese medicine institutions per 10,000 people (D2)
	Average number of medical technicians in traditional Chinese medicine hospitals per 10,000 people (D3)
Maternal and Child Health Care (E)	Prenatal examination rate (%) (E1)
	Postpartum visit rate (%) (E2)
	Delivery rate in hospital (%) (E3)
	Maternal mortality (%) (E4)
	Examination rate of gynecological diseases (%) (E5)
	Premarital examination rate (%) (E6)
People’s health and medical security (F)	Birth rate (%) (F1)
	Population mortality (%) (F2)
	life expectancy (F3)
	Average number of health education trainers per 10,000 people (F4)
	Accumulated balance of basic medical insurance for urban employees (CNY) (F5)
Disease Control and Public Health (G)	Incidence rate of Class A and B notifiable infectious diseases (%) (G1)
	Class A and B notifiable infectious diseases report infectious disease mortality (%) (G2)
	Rural population of drinking water accounts for rural population (%) (G3)
	Prevalence rate of sanitary toilets in rural areas (%) (G4)

### Research methods

1. **Entropy method** The entropy weight method is a technique that utilizes entropy values to assess the degree of variation among indicators, determine the weights of indicators, and measure the amount of information provided by data [42]. The entropy method is considered one of the objective weighting methods. This approach determines the weight of each index based on the actual situation of the data, thereby avoiding any biases caused by dominant factors.
  It is important to note that the entropy method relies entirely on the completeness and accuracy of the collected data. Therefore, it is crucial to ensure that the data used in the analysis is both comprehensive and precise.
  Furthermore, the entropy method determines the weights of indicators by assessing local differences. As a result, it is commonly employed for the relative evaluation of multiple indicators, allowing for a comprehensive assessment of their relative importance.
2. **Difference analysis method based on Theil index** Theil index, also known as the Theil’s entropy measure, was proposed by H.Theil, a Dutch economist, in 1967 to measure the income gap between individuals or regions [43]. The range of Thiel index is 0 ~ 1. A large value indicates a large regional difference, while a large value indicates a small regional difference [44].
3. **Convergence analysis** The term “convergence” originates from the field of mathematics, describing the behavior of the terms in a sequence approaching a certain limit value. In economics, this concept is introduced to explore the dynamic behavior of different economies under specific conditions, particularly the study of the negative correlation between the initial state of economic entities and their growth rate within a given effective range [45]. Economists frequently employ concepts such as sigma convergence (*σ*-convergence) and beta convergence (*β*-convergence) to analyze this phenomenon.
  In this context, σ-convergence refers to the assessment of convergence by analyzing the distribution of the standard deviation of a certain variable across regions [46]. β-convergence is further divided into absolute β-convergence and conditional β-convergence. Absolute β-convergence suggests that economies with identical economic structures will, over time, converge towards the same path of economic growth and eventually stabilize at a uniform state. Conditional β-convergence indicates that economies with different economic structures, influenced by external factors, will converge towards their respective steady-state levels [47].
  In this study, σ-convergence specifically refers to the diminishing disparities in the scores of basic medical and health service level evaluations among different regions over time. Common statistical methods to measure σ-convergence include the Gini coefficient, Theil index, and the Coefficient of Variation (CV). In this paper, we have employed the CV, which is widely used in many research studies, as a tool to measure σ-convergence.
  In this study, the convergence of the level of basic medical and health services in China was investigated by *β* convergence form. *β* convergence is derived from the theory of economic convergence in neoclassical economy and was used in the early discussion of economic growth convergence between regions or countries [47–49].

### Data source and research area

1. **Data source and description** Since the 18th National Congress of the CPC (2012), the government has given priority to ensuring people’s health, and integrated medical reform into the overall deepening of reform [1]. China’s health care has developed rapidly. Therefore, the starting point of this study is 2012. Due to the serious lack of some data in China’s provinces and regions from 2020 to now, the time range of this study is finally determined as 2012–2019.
  The data of this study were collected from China Statistical Yearbook, China Health Statistical Yearbook, China Finance Yearbook, China Rural Statistical Yearbook, National Statistics on Traditional Chinese Medicine, Statistical Yearbook of each province, and Statistical Bulletin of health development of each province. The individual data of Tibet Autonomous Region came from authoritative websites. The average life expectancy data of each province from 2012 to 2019 is calculated from the data of the sixth national population census in 2010 and the data of the bulletin of the seventh national population census published by each province in 2020. The calculation formula is as follows:
$$\overline {{{A_n}}} =\overline {{{A_{n - 1}}}} +\frac{{(\overline {{{A_{2020}}}} - \overline {{{A_{2010}}}} )}}{{10}}$$
  Where $\overline {A}$ is the average life expectancy, n is the year, *n* = 2011, 2012,…, 2019.
  Among them,average life expectancy is calculated by applying the concept of hypothetical cohorts along with the life table approach to estimate the average age at which death is expected to occur [50].
2. **Description of regional division** According to the Division Method of East, West, Central, and Northeast Regions published on the official website of the National Bureau of Statistics in 2011, this paper divides 31 provinces (municipalities and autonomous regions) into four regions: northeast, east, central and west,. Among them, the northeastern region includes Liaoning Province, Jilin Province and Heilongjiang Province. The eastern region includes Beijing Municipality, Tianjin Municipality, Hebei Province, Shanghai Municipality, Jiangsu Province, Zhejiang Province, Fujian Province, Shandong Province, Guangdong Province and Hainan Province. The central region includes Shanxi Province, Anhui Province, Jiangxi Province, Henan Province, Hubei Province and Hunan Province. The western region includes Inner Mongolia Autonomous Region, Guangxi Province, Chongqing Municipality, Sichuan Province, Guizhou Province, Yunnan Province, Tibet Autonomous Region, Shaanxi Province, Gansu Province, Qinghai Province, Ningxia Autonomous Region and Xinjiang Autonomous Region.

## Results

### Analysis of the measurement results of basic medical and health services in China

**Fig. 1:**
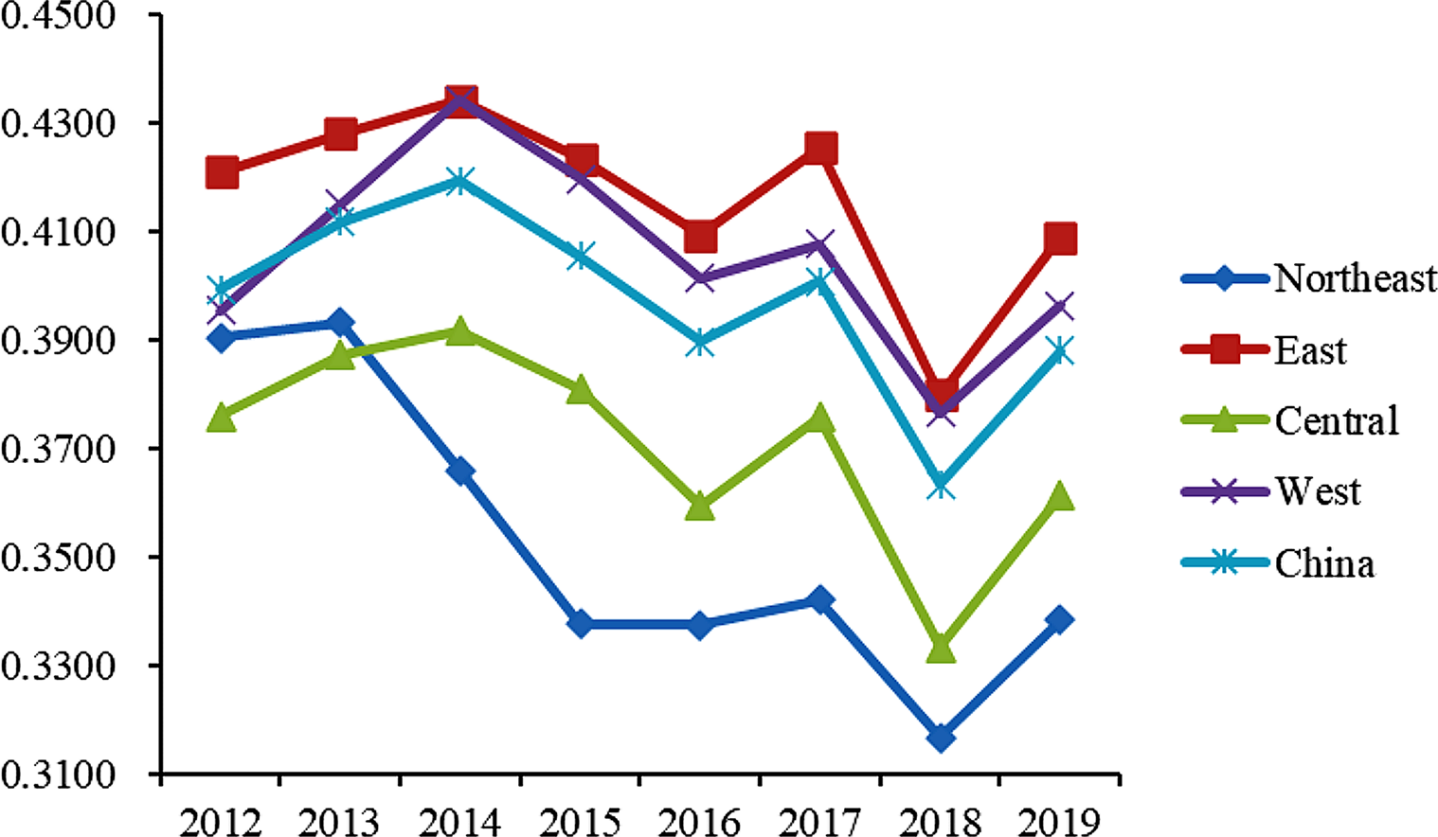
Average scores of basic medical and health services in China and the four major regions (2012–2019)

As depicted in Table 2, the weight assigned to each index from 2012 to 2019 was determined using the entropy method employed in this study. Moreover, Table 3 showcases the computed scores of basic medical and health services for all provinces and regions of China spanning the years 2012 to 2019. Subsequently, the average scores for the provinces (cities and autonomous regions) were calculated separately for China as a whole and the four main regions. To provide a visual representation of the research findings and facilitate a clear and intuitive observation of the changes in the level of basic medical and health services in the study area, a line graph has been employed (refer to Fig. 1).

**Table 2:** Weights of the evaluation indicators for the level of basic medical and health services in China (2012–2019)

	2012	2013	2014	2015	2016	2017	2018	2019
**A1**	0.0428	0.0379	0.0367	0.0370	0.0345	0.0332	0.0294	0.0306
**A2**	0.0300	0.0307	0.0310	0.0293	0.0279	0.0277	0.0255	0.0277
**A3**	0.0686	0.0441	0.0439	0.0450	0.0555	0.0635	0.0583	0.0644
**A4**	0.0233	0.0733	0.0321	0.0357	0.0309	0.0332	0.0348	0.0381
**A5**	0.0592	0.0526	0.0498	0.0557	0.0569	0.0506	0.0504	0.0462
**A6**	0.0373	0.0385	0.0389	0.0383	0.0376	0.0379	0.0358	0.0389
**B1**	0.0400	0.0423	0.0479	0.0431	0.0376	0.0343	0.0239	0.0245
**B2**	0.0083	0.0087	0.0080	0.0077	0.0079	0.0083	0.0073	0.0073
**B3**	0.0349	0.0347	0.0360	0.0361	0.0383	0.0393	0.0383	0.0424
**B4**	0.0281	0.0197	0.0143	0.0142	0.0202	0.0243	0.0298	0.0182
**B5**	0.0170	0.0175	0.0269	0.0283	0.0206	0.0235	0.0204	0.0241
**B6**	0.0082	0.0079	0.0080	0.0076	0.0074	0.0068	0.0065	0.0072
**B7**	0.0091	0.0095	0.0094	0.0091	0.0091	0.0092	0.0084	0.0094
**C1**	0.0159	0.0177	0.0160	0.0151	0.0167	0.0155	0.0162	0.0179
**C2**	0.0138	0.0185	0.0175	0.0178	0.0196	0.0334	0.0372	0.0392
**C3**	0.0305	0.0284	0.0253	0.0180	0.0165	0.0180	0.0165	0.0181
**C4**	0.0138	0.0193	0.0292	0.0290	0.0236	0.0333	0.0247	0.0223
**C5**	0.0128	0.0128	0.0131	0.0113	0.0108	0.0096	0.0087	0.0095
**C6**	0.0309	0.0389	0.0424	0.0415	0.0277	0.0224	0.0226	0.0238
**D1**	0.0416	0.0383	0.0407	0.0398	0.0424	0.0436	0.0367	0.0399
**D2**	0.0245	0.0345	0.0394	0.0357	0.0316	0.0295	0.0280	0.0277
**D3**	0.0356	0.0322	0.0312	0.0295	0.0364	0.0340	0.0286	0.0271
**E1**	0.0070	0.0061	0.0114	0.0077	0.0094	0.0103	0.0052	0.0061
**E2**	0.0072	0.0075	0.0082	0.0101	0.0138	0.0099	0.0054	0.0061
**E3**	0.0056	0.0058	0.0058	0.0057	0.0056	0.0055	0.0049	0.0057
**E4**	0.0056	0.0058	0.0062	0.0060	0.0056	0.0057	0.0063	0.0061
**E5**	0.0289	0.0318	0.0224	0.0215	0.0177	0.0218	0.0088	0.0087
**E6**	0.0472	0.0437	0.0442	0.0389	0.0356	0.0386	0.0329	0.0329
**F1**	0.0200	0.0207	0.0266	0.0324	0.0215	0.0252	0.0249	0.0269
**F2**	0.0501	0.0358	0.0420	0.0389	0.0418	0.0367	0.0275	0.0346
**F3**	0.0159	0.0158	0.0156	0.0148	0.0141	0.0138	0.0128	0.0140
**F4**	0.0479	0.0476	0.0553	0.0779	0.1040	0.0680	0.1593	0.1140
**F5**	0.0700	0.0745	0.0794	0.0781	0.0783	0.0837	0.0791	0.0862
**G1**	0.0084	0.0085	0.0079	0.0074	0.0075	0.0088	0.0070	0.0144
**G2**	0.0071	0.0081	0.0085	0.0092	0.0095	0.0109	0.0126	0.0118
**G3**	0.0368	0.0148	0.0125	0.0124	0.0123	0.0130	0.0135	0.0172
**G4**	0.0161	0.0157	0.0162	0.0141	0.0138	0.0170	0.0122	0.0109

*Note* For detailed descriptions corresponding to the indicator abbreviations from A1 to G4, please refer to Table 1

**Table 3:** Scores of basic medical and health services of provinces (municipalities, autonomous regions) in China (2012–2019)

Region	Province	2012	2013	2014	2015	2016	2017	2018	2019	Provincial average score in 2012–2019	Regional average score in 2012–2019
Northeastern China	Heilongjiang	0.3997	0.4163	0.3951	0.3577	0.3338	0.3367	0.3048	0.3227	0.3584	0.3528
	Jilin	0.3954	0.3804	0.3483	0.3432	0.3537	0.3527	0.3424	0.3683	0.3606	
	Liaoning	0.3764	0.3832	0.3549	0.3120	0.3250	0.3369	0.3030	0.3241	0.3394	
Eastern China	Beijing	0.5215	0.5277	0.5142	0.4896	0.4663	0.4501	0.3902	0.4365	0.4745	0.4162
	Fujian	0.4022	0.4219	0.4340	0.4147	0.3749	0.3924	0.3481	0.3653	0.3942	
	Guangdong	0.4698	0.4730	0.4895	0.4911	0.4908	0.5114	0.4564	0.4912	0.4842	
	Hainan	0.3844	0.3546	0.3773	0.3605	0.3296	0.3590	0.3176	0.3433	0.3533	
	Hebei	0.3682	0.3872	0.4169	0.3989	0.3688	0.3997	0.3758	0.4110	0.3908	
	Jiangsu	0.4429	0.4416	0.4529	0.4358	0.4394	0.4426	0.4202	0.4421	0.4397	
	Shandong	0.4410	0.4687	0.4497	0.4351	0.4220	0.4511	0.4078	0.4404	0.4395	
	Shanghai	0.3898	0.3995	0.3802	0.3828	0.3736	0.3823	0.3282	0.3566	0.3741	
	Tianjin	0.3250	0.3224	0.3175	0.3190	0.3389	0.3603	0.3199	0.3405	0.3304	
	Zhejiang	0.4641	0.4831	0.5096	0.5061	0.4896	0.5046	0.4343	0.4631	0.4818	
Central China	Anhui	0.3469	0.3709	0.3812	0.3605	0.3455	0.3584	0.3325	0.3607	0.3571	0.3708
	Henan	0.3646	0.4010	0.3946	0.3841	0.3652	0.3780	0.3485	0.3726	0.3761	
	Hubei	0.3369	0.3490	0.3463	0.3652	0.3313	0.3349	0.2819	0.3052	0.3313	
	Hunan	0.3874	0.4217	0.4396	0.4327	0.3962	0.4213	0.3610	0.3832	0.4054	
	Jiangxi	0.3597	0.3456	0.3669	0.3475	0.3410	0.3780	0.3399	0.3785	0.3571	
	Shanxi	0.4606	0.4351	0.4222	0.3955	0.3779	0.3843	0.3366	0.3684	0.3976	
Western China	Gansu	0.4430	0.4573	0.5052	0.4974	0.5374	0.4789	0.4247	0.4406	0.4730	0.4058
	Guangxi	0.3477	0.3607	0.3965	0.3902	0.3823	0.3924	0.3547	0.3713	0.3745	
	Guizhou	0.3075	0.3483	0.3768	0.3553	0.3295	0.3404	0.3097	0.3305	0.3372	
	Inner Mongolia	0.4350	0.4688	0.4757	0.4554	0.4281	0.4560	0.3919	0.4428	0.4442	
	Ningxia	0.4323	0.4473	0.4795	0.4364	0.4518	0.4274	0.4459	0.4041	0.4406	
	Qinghai	0.4066	0.4123	0.4631	0.4679	0.3803	0.4505	0.5115	0.4968	0.4486	
	Shaanxi	0.3807	0.4333	0.4226	0.4027	0.3910	0.3788	0.3234	0.3571	0.3862	
	Sichuan	0.4107	0.4624	0.4589	0.4337	0.4175	0.4213	0.3698	0.4012	0.4219	
	Tibet	0.4542	0.4329	0.4453	0.4631	0.4005	0.4422	0.4159	0.4669	0.4401	
	Xinjiang	0.4763	0.4698	0.4776	0.4547	0.4444	0.4276	0.3656	0.3792	0.4369	
	Yunnan	0.3253	0.3695	0.3781	0.3591	0.3395	0.3608	0.3114	0.3498	0.3492	
	Chongqing	0.3257	0.3181	0.3300	0.3212	0.3133	0.3162	0.2937	0.3172	0.3169	

*Note* This study employed the entropy method to calculate the scores of the basic medical and health service levels for each province in China and the four major regions from 2012 to 2019

**Fig. 3:**
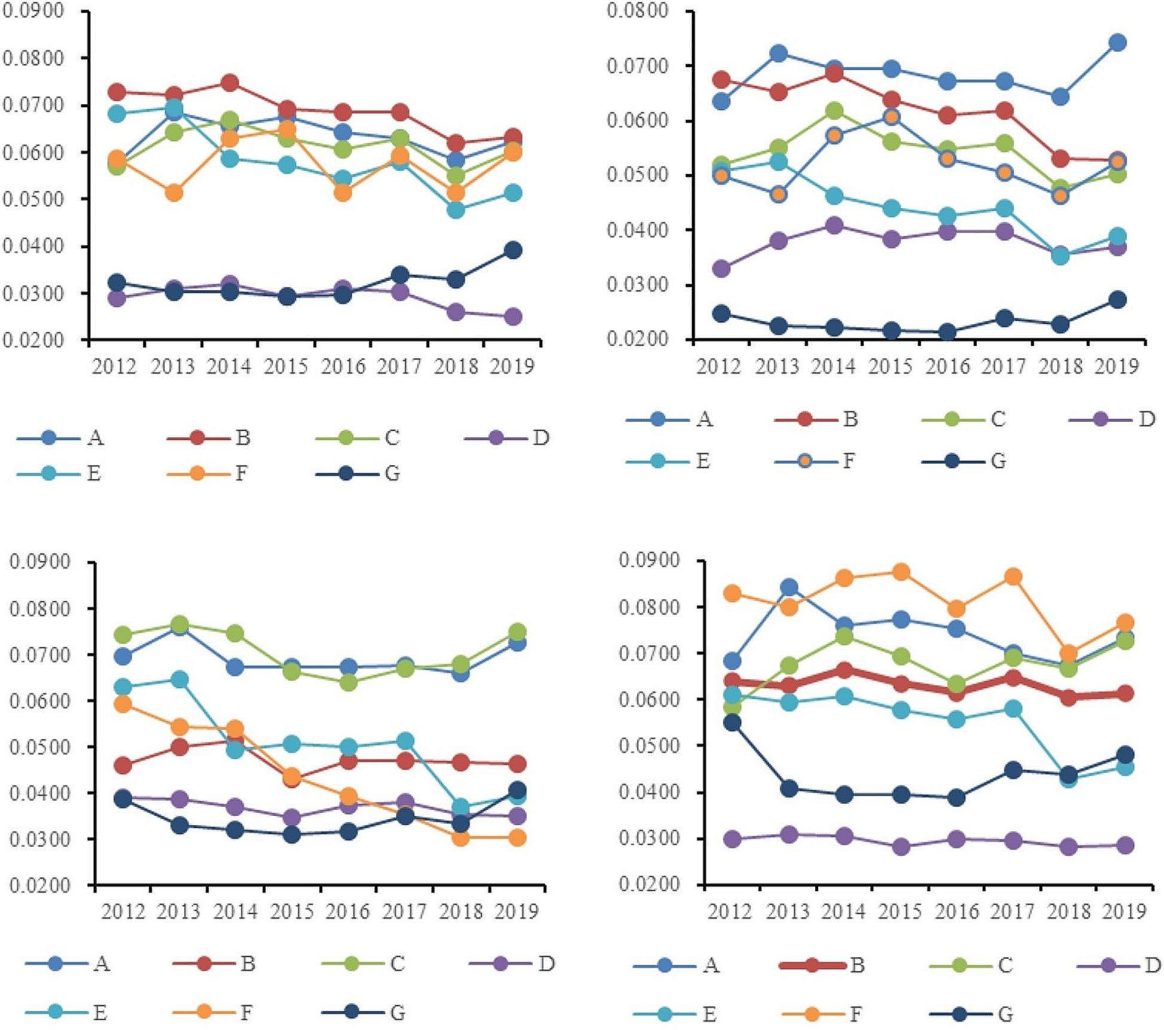
Scores of various dimensions of basic medical and health service levels in China’s four major regions (2012–2019). (**a**) Northeast region, (**b**) Eastern region, (**c**) Central region, (**d**) Western region

1. **National level** During the period of 2012–2019, the score range for China’s basic medical and health services was observed to be between 0.36 and 0.42 (As shown in Fig. 1). It is evident that the overall level of these services was not particularly high, leaving ample room for improvement. As depicted in Fig. 1, a distinctive “W” shaped change trend can be observed, with relatively minor fluctuations. The average interval score calculated was 0.3973, indicating a general stability in China’s basic medical and health services. From an indicator perspective, it is noteworthy that within this range, most evaluation indicators demonstrated some degree of improvement. Furthermore, all dimensions of the basic medical and health service level in China have shown development, indicating enhanced coordination and balance across these dimensions.
  As illustrated in Fig. 2, during the period from 2012 to 2019, China’s basic medical and health service level experienced relatively stable development across multiple dimensions, with the exception of maternal and child health care. Specifically, in terms of medical and health facilities, the highest scores were achieved within the aforementioned time frame, with an average score of 0.0706, indicating that China has made more significant progress in the construction of medical and health facilities compared to other dimensions.
  **Fig. 2:**
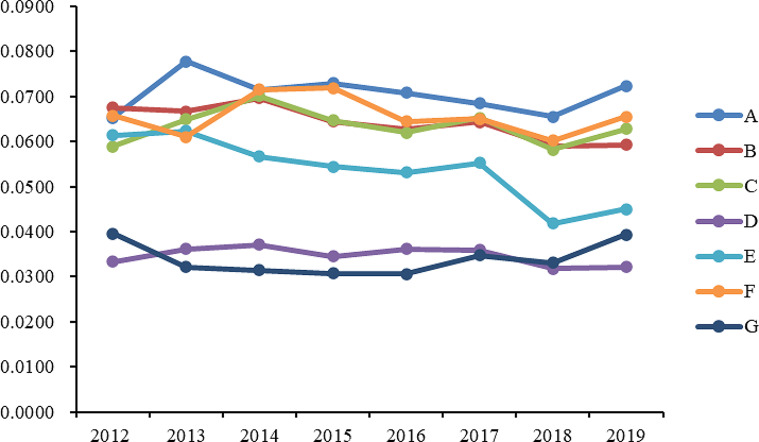
Scores of Various Dimensions of National Basic Medical and Health Service Levels (2012–2019). *Note *** A** represents Medical and Health Facilities, **B** represents Health Expenditure, **C** represents Medical Service, **D** represents Traditional Chinese Medicine Hospital Services, **E** represents Maternal and Child Health Care, **F** represents People’s Health and Medical Security, **G** represents Disease Control and Public Health
  Regarding the dimension of health funding, the average score was 0.0642, exhibiting a fluctuating trend. In the dimension of medical services, the average score was 0.0634, with no significant fluctuations observed in the various indicators within this dimension. In the dimension of maternal and child health care, scores were relatively stable from 2012 to 2017, with fluctuations ranging between 0.5300 and 0.6300. In the dimension of public health and medical security, the average scores from 2012 to 2019 remained at a high level with minor fluctuations. However, in 2018, there was a significant drop in this dimension, mainly reflected in the decrease in the birth rate, which dropped to an average of 10.94% for the year, while from 2012 to 2017, the average birth rate was maintained at about 12%. As for the dimension of disease control and public health, although its average score was the lowest among the seven dimensions, there has been an increase in scores since 2016.
2. **Regional level** As illustrated in Fig. 1, regarding the change trend of basic medical and health service scores from 2012 to 2019 in the four major regions of China, a similar pattern emerges. The eastern region stands out with the highest overall score, averaging at 0.4162. Following closely, the western region exhibits a slightly lower medical and health service level, receiving an evaluation score of 0.4058. Ranking third in terms of medical and health service level is the central region, with an average score of 0.3708. Lastly, the northeastern region trails behind with an average score of 0.3528. Evidently, the eastern region surpasses the other three regions in terms of basic medical and health services. This region encompasses economically developed provinces (municipalities and autonomous regions) such as Beijing, Tianjin, Hebei, the Yangtze River Delta, and the Pearl River Delta. Evaluation data suggests that the eastern region boasts comprehensive medical and health facilities, a high per capita allocation of such facilities, substantial government health expenditure, and a noteworthy improvement in average life expectancy. In the northeastern region, certain indicators of basic medical and health services have witnessed improvement. Notably, medical and health facilities, medical services, traditional Chinese medicine hospital services, and maternal and child health care dimensions perform at a middling to upper level nationwide. However, the dimensions of health expenditure, people’s health, and medical security lag slightly behind. Upon analyzing specific indicators, it becomes evident that government health expenditure and birth rates in the northeastern region are significantly lower compared to other regions.
  As shown in Fig. 3, we observe that the trends and amplitude of fluctuations across various dimensions show noticeable regional differences in the four major regions. In the Northeast region, as shown in Fig. 3(a), scores across the seven evaluation dimensions are relatively balanced. Among them, scores in the dimensions of medical health facilities and medical services are relatively prominent. Conversely, the scores in the dimension of public health and medical security are not only relatively low but also show a decreasing trend year by year. Through specific indicator analysis, we find that the birth rate in the Northeast region is significantly lower than other provinces, which may be a key factor in the score reduction. In the Eastern region, as illustrated in Fig. 3(b), the scores for the dimension of traditional Chinese medicine services are significantly lower than other dimensions. This phenomenon is reflected in specific indicators, such as the number of traditional Chinese medicine institutions and the number of beds per ten thousand people being relatively low. However, scores in other dimensions for this region are usually among the top two in the four major regions. As depicted in Fig. 3(c), the Central region shows greater volatility and an overall downward trend from 2012 to 2018, but scores in most dimensions rebounded in 2019. Overall, scores across various dimensions in the Central region are fairly balanced, with the dimensions of traditional Chinese medicine services and disease control and public health scoring lower, while fluctuations in other dimensions range between 0.4700 and 0.7300. For the Western region, as shown in Fig. 3(d), the distribution of scores across dimensions is more dispersed, with the average score for the dimension of disease control and public health being the lowest at 0.0228, and the dimension of medical health facilities scoring the highest on average at 0.0677. This result highlights the uneven development across different public health dimensions in the Western region.

### Difference analysis of China’s basic medical and health service level based on the Theil index

The calculation formula of the Theil index was applied to determine the Theil index of basic medical and health service levels in the four major regions of China from 2012 to 2019. Additionally, the Theil index within and between regions was computed, as shown in Table 4. Furthermore, the contribution rate of the Theil index in each of the four major regions was calculated and presented in Table 5.

**Table 4:** Theil index of basic medical and health service levels in four major regions of China (2012–2019)

Year	T	T^W^ Northeastern China	T^W^ Eastern China	T^W^ Central China	T^W^ Western China	T^W^	T^B^
2012	0.2962	0.0220	0.2539	0.0689	0.4070	0.2569	0.0393
2013	0.2729	0.0170	0.2326	0.0493	0.3645	0.2307	0.0421
2014	0.2823	0.0162	0.2257	0.0480	0.3764	0.2362	0.0461
2015	0.2866	0.0253	0.2231	0.0432	0.3905	0.2415	0.0451
2016	0.2740	0.0265	0.2191	0.0452	0.3615	0.2287	0.0453
2017	0.2764	0.0235	0.2175	0.0459	0.3811	0.2348	0.0417
2018	0.3057	0.0320	0.2067	0.0432	0.4462	0.2590	0.0467
2019	0.2897	0.0333	0.2108	0.0467	0.4139	0.2466	0.0431

*Note* T, Total Thiel index; T^W^, Intra- regional Theil index; T^B^, Inter- regional Theil index

**Table 5:** The contribution rate of thiel index of basic medical and health service level in four major regions of China

Year	T^W^ Northeast	T^W^ East	T^W^ Central	T^W^ West	T^W^(Intra-regional)	T^B^(Inter-regional)
2012	0.70%	29.14%	4.24%	52.65%	86.73%	13.27%
2013	0.58%	28.57%	3.29%	52.12%	84.56%	15.44%
2014	0.48%	26.69%	3.07%	53.42%	83.67%	16.33%
2015	0.71%	26.22%	2.74%	54.61%	84.28%	15.72%
2016	0.81%	27.11%	2.94%	52.60%	83.47%	16.53%
2017	0.70%	26.93%	3.01%	54.27%	84.92%	15.08%
2018	0.88%	22.80%	2.51%	58.53%	84.72%	15.28%
2019	0.97%	24.74%	2.90%	56.50%	85.11%	14.89%

*Note* T, Total Thiel index; T^W^, Intra- regional Theil index; T^B^, Inter- regional Theil index

#### Calculation and analysis of the overall differences in the level of basic medical and health services in China

**Fig. 4:**
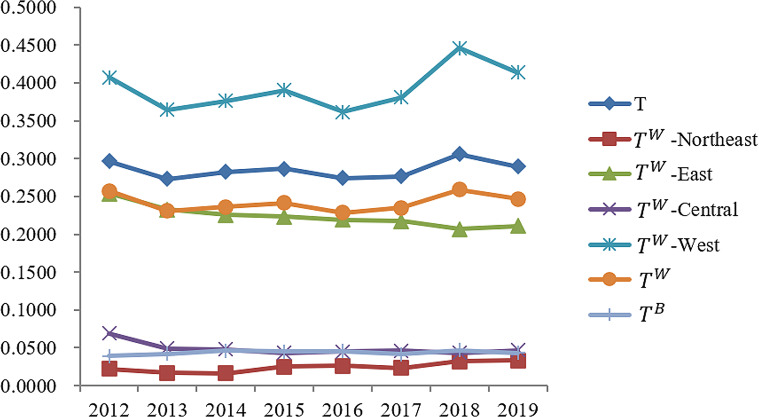
Thai Index of basic medical and health services in four regions of China (2012–2019). T, Total Thiel index; T^w^-Northeast, Intra-regional Thai index about northeastern region; Tw-East, Intra-regional Thai index about eastern region; T^w^-Central, Intra-regional Thai index about central region; T^w^-West, Intra-regional Thai index about western region; T^w^, Intra-regional Thai index; T^B^, Inter-regional Thai index

According to the calculation results of the Theil index, significant regional disparities are evident in the level of basic medical and health services in China. The maximum score for basic medical and health services was 0.3057, observed in 2018, while the minimum score was 0.2729, observed in 2013. The average score was 0.2855. Figure 4 illustrates the slight changes in the Theil index of the basic medical and health service level in China from 2012 to 2019, without any significant increases or decreases. This suggests that the regional disparities in the basic medical and health service level in China have not undergone any significant changes.

#### Comparison and analysis of the differences in the level of basic medical and health services in four major regions of China

The intra-regional (*T*^*W*^) Theil index for basic medical and health services in China from 2012 to 2019 was significantly higher than the inter-regional index (*T*^*B*^). Moreover, both indexes experienced minimal changes during this period. Over the specified timeframe, the intra-regional Theil index contributed more than 83% to the overall Theil index, while the inter-regional index contributed less than 17%. This observation suggests that the differences within regions in the level of basic medical and health services in China were greater than the differences between regions.

Breaking down the contributions by region, the Theil index accounted for 52 to 59% in the western region, 22 to 28% in the eastern region, 2.5 to 3.1% in the central region, and less than 0.1% in the northeastern region. Notably, the actual value of the Theil index in the western region was the highest, indicating that the disparity in the medical and health service level between provinces (municipalities and autonomous regions) in the western region was the largest, followed by the eastern, central, and northeastern regions.

### Convergence analysis of basic medical and health services in China

#### Analysis of ***σ*** convergence in the evaluation scores of basic medical and health service levels

**Fig. 5:**
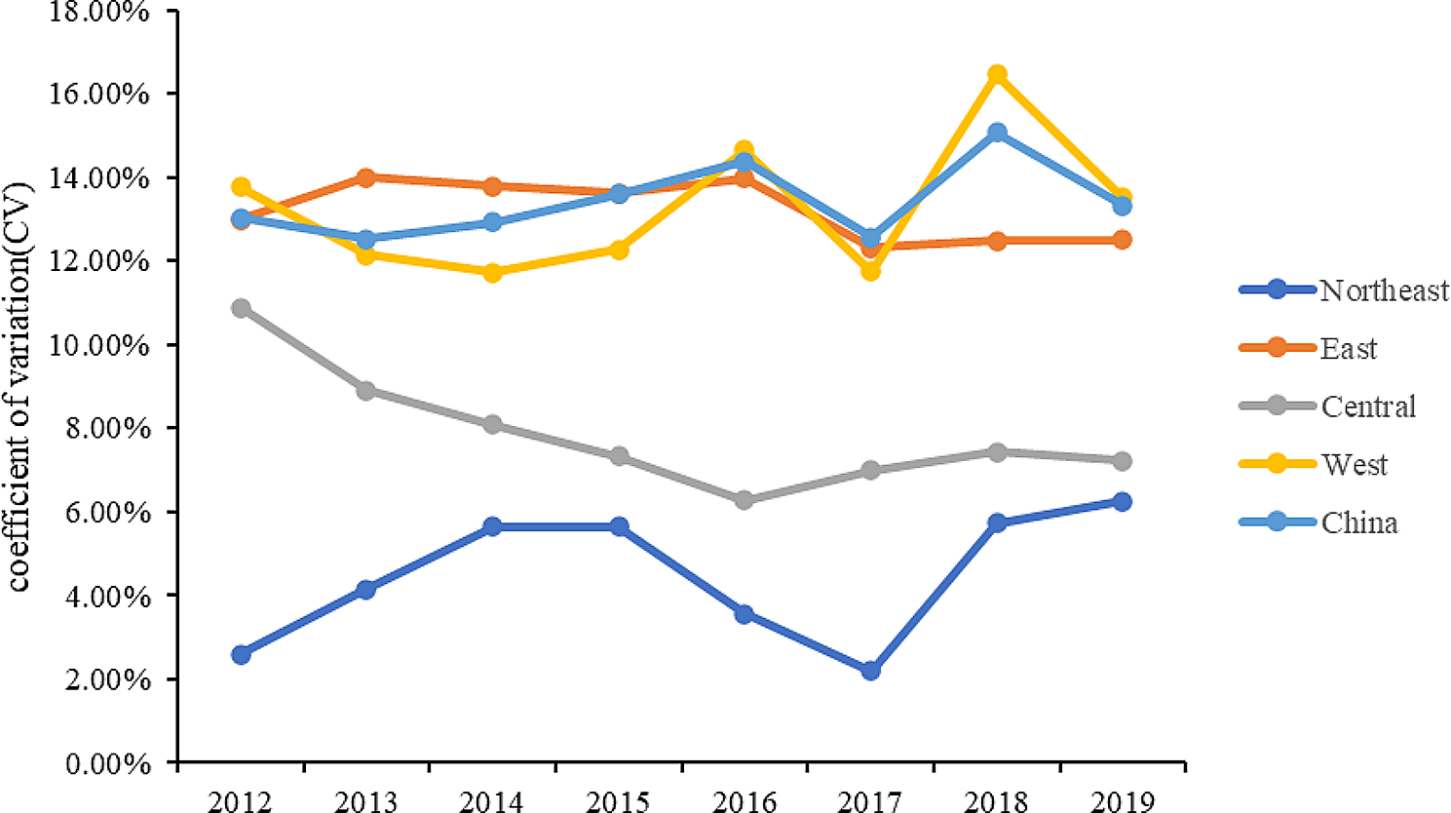
The σ-convergence trend in the level of basic medical and health services in China

In this study, we examine the disparities in the evaluation scores of basic medical and health service levels across different regions in China and investigate the trends of these disparities over time, namely, the phenomenon of *σ* convergence. *σ* convergence refers to the gradual narrowing of the gap in service level evaluation scores between regions over time. To measure this convergence trend, we employed several common sigma convergence calculation methods, including the Gini coefficient, Theil index, and Coefficient of Variation (CV). Among these, the Coefficient of Variation, due to its widespread use in existing research literature [51–52], was selected as the main analytical tool for this paper. Based on data from 2012 to 2019, we calculated *σ* convergence indices for China’s northeastern, eastern, central, and western regions. As shown in Fig. 5, by presenting these indices in a line chart, we found that the basic medical and health service levels in China did not exhibit a consistent *σ* convergence trend. Specifically, the northeastern region displayed a pattern of “divergence-convergence-divergence”; the eastern region showed a convergence trend from 2012 to 2017, but a divergence from 2018 to 2019; the central region demonstrated a convergence trend from 2012 to 2016, but shifted to a “divergence-convergence” pattern from 2017 to 2019; the western region exhibited alternating characteristics of convergence and divergence. These complex trends indicate that during the examined period, China and its four major regions did not exhibit significant *σ* convergence in the level of basic medical and health services.

#### Analysis of Absolute ***β*** convergence in the evaluation scores of basic medical and health service levels

As presented in Table 6, the regression analysis employed three models: Mixed-effect, Fixed-effect, and Random-effect models. The regression results for the absolute *β* convergence test are displayed. Upon examining the regression outcomes, it is evident that all three regression models yield identical results. The *β* coefficients of absolute convergence for China’s four major regions are all below 0 and have passed the significance test at the 1% or 5% level. This suggests a negative relationship between the level of basic medical and health services in these regions and their initial characteristics, indicating the existence of absolute convergence. In essence, disregarding regional heterogeneity, the level of basic medical and health services in all provinces, municipalities, and autonomous regions in China will eventually converge steadily to the same level. The Fixed-effect model estimates the *β* values for the convergence speeds of the four main regions in China as follows: 0.1235, 0.0551, 0.1386, and 0.1344, respectively. These figures indicate that the western region exhibits the fastest convergence speed, while the northeastern region demonstrates the slowest rate of convergence.

**Table 6:** Absolutely *β* Convergence test of basic medical and health service level in China

Model Type	Items	China	Northeastern China	Eastern China	Central China	Western China
Mixed-effect	*β*	-0.1358***	-0.3244**	-0.1130**	-0.3291***	-0.1714***
		(0.0346)	(0.1452)	(0.0549)	(0.1099)	(0.0602)
	Constant	-0.1304***	-0.3581**	-0.1039**	-0.3321***	-0.1553***
		(0.0326)	(0.1515)	(0.0492)	(0.1096)	(0.0554)
	R-squared	0.0624	0.1665	0.0448	0.1831	0.0900
	obs	217	21	70	42	84
Fixed-effect	β	-0.6276***	-0.3563**	-0.6701***	-0.6587***	-0.6921
		(0.0670)	(0.1573)	(0.1209)	(0.1534)	(0.1150)
	Constant	-0.5876***	-0.3912**	-0.5963***	-0.6594***	-0.6284***
		(0.0625)	(0.1641)	(0.1071)	(0.1527)	(0.1048)
	R-squared	0.3214	0.2318	0.3424	0.3450	0.3377
	obs	217	21	70	42	84
Random-effect	*β*	-0.1358***	-0.3244**	-0.1130**	-0.3291***	-0.1714***
		(0.0346)	(0.1452)	(0.0549)	(0.1099)	(0.0602)
	Constant	-0.1304***	-0.3581**	-0.1039**	-0.3321***	-0.1553***
		(0.0326)	(0.1515)	(0.0492)	(0.1096)	(0.0554)
	R-squared	0.0668	0.2081	0.0586	0.1831	0.0900
	obs	217	21	70	42	84

*Note* ***, **, and * represent significance at the 1, 5, and 10% levels, respectively

The convergence test results are in line with the development of basic medical and health services in China during 2012–2019. On September 7, 2022, the Propaganda Department of the Central Committee of CPC held a series of press conferences on “China’s Decade” to highlight the achievements of health development since the 18th National Congress of the CPC. During the press conference, it was mentioned that since 2012, the Chinese government has implemented the health poverty alleviation project and successfully ensured basic medical security for the rural poor. By the end of 2020, over 20 million individuals had been saved and cured. Additionally, the per capita subsidy for basic public health services was increased to 84 yuan. Health management services were provided for patients with key diseases such as hypertension, diabetes, and tuberculosis, as well as for specific groups such as children aged 0–6 years, pregnant women, and individuals over 65 years old [38]. Moreover, the Chinese government has enhanced the capacity of county-level hospitals and progressively reduced the disparity in medical service capacity between urban and rural areas.

## Main conclusions and suggestions

### Main conclusions

This study develops an evaluation index system for basic medical and health services that aligns with China’s national conditions. Using provincial panel data from 2012 to 2019, the study employs the panel entropy method to calculate the level of basic medical and health services in China. Furthermore, the study utilizes the Theil index, β convergence test, and other methods to analyze the regional differences and convergence characteristics of the level of basic medical and health services. The main conclusions are as follows:

1. Between 2012 and 2019, the score range for China’s basic medical and health service level fell between 0.36 and 0.42, with an average score of 0.3973. In analyzing the scores of basic medical and health service levels among various provinces in China, we noticed that the scores of some provinces are relatively low, which differs from the conclusions of Dong et al. [53], who suggested that the service equality in the three western provinces of Guizhou, Qinghai, and Tibet is the lowest. To explore the differences in evaluation results, this paper conducts further analysis. In terms of the selection of indicators for medical input, Dong et al. [53] referred to the total expenses of medical and health institutions, while this paper adopts per capita expenses and introduces the indicator of the proportion of total health expenses to GDP. Considering that the population density in the western region is relatively lower than that in the eastern and central regions, the use of per capita indicators may affect the evaluation results. This is because it may amplify the input of medical resources in regions with smaller populations, leading to a deviation between the evaluation results and the actual service equality. Therefore, the choice of evaluation indicators and the differences in regional characteristics may both be reasons for the discrepancies in evaluation results.
2. The calculation results of the Theil index indicate that between 2012 and 2017, there were significant regional disparities in the level of basic medical and health services in China. Intra-regional differences were more pronounced compared to inter-regional differences. The findings of this study show that the trend of changes in the basic medical and health service levels of various provinces in China is similar to the study by Yu et al. [54], who noticed a downward turning point in 2017 when analyzing the efficiency of China’s medical and health service system from 2010 to 2017. This result is supported by the research of Liu et al. [55], who found that the annual growth rate of the comprehensive evaluation index for medical and health services in the eastern, central, and western regions of China showed a trend of eastern < central < western regions, while the annual growth rate of the comprehensive economic evaluation index was central < eastern < western. This indicates that the medical and health service evaluation index in the western region has not only improved but also increased at a rate surpassing other regions. What is more noteworthy is that the level of medical and health services in most provinces in the central and western regions has advanced beyond their level of economic development. These conclusions are not only consistent with our research results but also further point out the possible reasons for regional differences. The rapid development of medical and health services in the central and western regions may benefit from policy support and the prioritization of resource allocation. Their development speed has even surpassed the pace of economic growth, providing an important reference for formulating differentiated regional development strategies.
3. During the research period from 2012 to 2019, neither China as a whole nor its four major regions, the Northeast, East, Central, and West, showed a significant trend of *σ*-convergence, which differs from the findings of Xin et al. [36]. There are mainly two reasons for this discrepancy: firstly, the time intervals selected for the studies are different; secondly, the indicator system used in this study has been enriched in both dimensions and the diversity of indicators. However, in terms of *β*-convergence, China and its four regions have shown a clear trend, indicating that the disparities in the basic medical and health service levels among the provinces are narrowing, and there is an emerging pattern of provinces with lower service levels catching up with those with higher levels. This finding suggests that although the absolute differences in service levels have not been completely eliminated, there is a dynamic equilibrium process in the improvement of basic medical and health services between regions, which is of great significance for understanding and assessing the balanced development of health services between regions in China.

### Policy recommendations

Based on the above conclusions, this paper proposes the following suggestions:

The central government should explore and leverage the advantages of Internet-based medical services to enhance interactions among provinces within and across regions, provide quality medical services to the public, and narrow the gap in basic medical services between provinces within and across regions as well as between urban and rural areas. Simultaneously, it is suggested that the government or relevant departments should establish a comprehensive standard for “Internet plus medical care”, innovate the service and payment modes, and enhance the transparency of Internet-based medical care.

In order to promote the unique characteristics of Traditional Chinese Medicine (TCM) and enhance the utilization rate of Chinese medicinal materials, it is necessary to increase policy and resource allocation support for TCM and TCM hospitals. This means not only increasing the number of TCM hospitals and expanding the scale of TCM hospital beds but also enhancing the training of Chinese medicine practitioners to grow the team of TCM professionals. Furthermore, active promotion of the application of TCM in the treatment of common diseases should be undertaken, and the unique advantages of TCM in primary medical care, physiotherapy, and the prevention and treatment of pre-disease conditions should be fully utilized. Through these comprehensive measures, TCM can be more effectively integrated into the modern medical service system, enriching the content of medical services and providing new pathways to improve public health levels.

To strengthen the prevention and control of infectious diseases, it is crucial to enhance public awareness and vigilance against infectious diseases, which requires sustained public education and promotion efforts. At the same time, building a professional and high-quality public health workforce is essential, as it directly strengthens the emergency response and management capabilities for public health incidents. Moreover, fortifying the construction of the disease prevention management system and increasing fiscal support for grassroots public health initiatives will provide a solid foundation for disease prevention and control. Establishing a comprehensive early warning and emergency response mechanism for infectious disease outbreaks, including decision-making, reporting, and response processes, is key to improving emergency handling capabilities. Through these measures, the occurrence and spread of infectious diseases can be effectively reduced, ensuring public health and safety.

China’s governments should enhance the capacity of medical and health services at the grassroots level, integrate resources, further improve the facilities of medical and health institutions at the grassroots level, and address the shortcomings of medical and health professionals at the grassroots level. Common and frequently occurring diseases among residents can be effectively addressed at the grassroots medical and health institutions, rather than accumulating cases of serious and minor diseases in the top three hospitals. This approach will help prevent the wastage of medical resources. China’s government should enable grassroots health and medical institutions to truly serve as the first line of defense for people’s health.

During the development process of various regions, the key to comprehensively enhancing the level of basic medical and health services lies in sustained efforts and focusing on filling the gaps in service capacity. This requires not only the continuous attention and efforts of local governments and relevant departments but also a close integration with national policies and guidelines. For instance, the Northeast region can rely on the “Fourteenth Five-Year Plan for the Comprehensive Revitalization of the Northeast” to promote improvements and innovations in medical services, while the Western region can strengthen the construction of medical and health infrastructure and service system improvement in accordance with the “Guiding Opinions on Promoting the Formation of a New Pattern in the Western Development in the New Era.” The Central region can similarly utilize the “Opinions of the CPC Central Committee and the State Council on Promoting High-Quality Development in the Central Region in the New Era” to optimize the allocation of medical resources and improve service quality and efficiency. Through such strategies, not only can balanced development in medical and health services be ensured across regions, but it can also help form a new pattern of integrated service capabilities that develops in coordination and improves collectively, further promoting the balanced development of medical and health services in all regions and the improvement of the health level of the population.

### Limitations of the Study and Future Outlook

The research methods and conclusions of this paper are expected to offer valuable references for the development of basic medical and health services in China. This paper utilizes provincial-level panel data in China. In the future, it is necessary to collect panel data at the city and county levels in China as much as possible for analysis, which would supplement and verify the conclusions of this study.

### Electronic supplementary material

Below is the link to the electronic supplementary material.

Supplementary Material 1

## Data Availability

The data and materials used to support the findings of this study are shared by the corresponding author on reasonable request.
