# Supplementary material for: Unveiling spatial disparities in basic medical and health services: insights from China’s provincial analysis
Source: BMC Health Serv Res. 2024 Mar 12;24:329. doi: 10.1186/s12913-024-10798-3 (PMC10936125; doi:10.1186/s12913-024-10798-3)
Supplement: Supplementary file 1 — Supplementary Material 1 [file 12913_2024_10798_MOESM1_ESM.docx]

**Appendix**

The research methods used in this paper include the Entropy method, the Theil index, and Convergence analysis. Below are the computational steps for each method:

**（1）Entropy method**

The specific calculation steps of panel entropy method are as follows:

Step 1: Standardize indicators. In order to eliminate the difference of indicator magnitude and unit, each indicator is standardized. The calculation formula is as follows:

Positive indicator:$X_{ijt}^{*}=\frac{x_{ijt}-\min(x_{i})}{\max(x_{i})-\min(x_{i})}$ （1）

Negative indicator:$X_{ijt}^{*}=\frac{\max(x_{i})-x_{ijt}}{\max(x_{i})-\min(x_{i})}$ （2）

Wherein, $x_{ijt}$ and $X_{ijt}^{*}$ represent the original value and standardized value of index *j* in the *t* year of the *i* province respectively, and $max(x_{i})$ and $min(x_{i})$ represent the maximum and minimum values of index *j* in the sample period of 31 provinces respectively.

Step 2: The entropy method is used to calculate the weight $W_{ij}$ of 37 indicators.

The principle of entropy method is to obtain the information entropy of each index according to the inherent information of each scheme in the evaluation. The larger the information entropy is, the smaller the utility value of information is, and the smaller the index weight is. The calculation process is as follows:

Calculate the proportion of the *j* index of the *i* province in the index in year *t*:

$Y_{ijt}=\frac{X_{ijt}^{*}}{\sum_{i=1}^{n} X_{ijt}^{*}}$，$i\in[1,n],j\in[1,m]$ （3）

Calculate the information entropy of the *j* index in year *t*:

$e_{jt}=-M\times\sum_{i=1}^{n} \left(Y_{ijt}\times\ln Y_{ijt}\right)$, $M=\frac{1}{\ln(n)}$ （4）

Calculate information entropy redundancy:

$d_{jt}=1-e_{jt}$ （5）

Calculate index weight:

$W_{jt}=\frac{d_{jt}}{\sum_{j=1}^{m} d_{jt}}$ （6）

Repeat steps 1-2 for each indicator to calculate the weight of all indicators for each year. The results are shown in Table 2.

Step 3: Measure the basic medical and health service level of each province in China.

First, calculate the standardized scores of 37 indicators in a year（$X_{ij}^{*}$）; Secondly, the weight of each index is multiplied by the standardized score of the corresponding index（$X_{ij}^{*}\times W_{j}$）； Finally, sum the weighted scores of each indicator, that is

$F_{i}=\sum_{j=1}^{37} (X_{ij}^{*}\times W_{j})$ （7）

Then the scores of medical and health service level of each province were obtained.

Repeat step 3 to calculate the scores of basic medical and health service level of each province in each year.

（2）Theil index

The calculation and decomposition formula of Thiel index is:

$T=\sum_{p=1}^{n} \frac{y_{p}}{Y}\log\left[\left(\frac{y_{p}}{Y}\right)/\left(\frac{1}{n}\right)\right]$ （8）

where $y_{p}$ is income of person indexed by *p*, n is the number of individuals in the population and *Y* representing the population’s total income, $Y=\sum_{p=1}^{n} y_{p}$.

Theil index can be decomposed into between group component (*T^B^*) and a within group component (*T^W^*). The decomposition formula is as follows:

$T^{_{B}}=\sum_{i=1}^{m} \frac{Y_{i}}{Y}\log\left[\left(\frac{Y_{i}}{Y}\right)/\left(\frac{n_{i}}{n}\right)\right]$ （9）

$T^{W}=\sum_{i=1}^{m} \frac{Y{}_{i}}{Y}T_{i}$ （10）

The Theil index for each group, *T_i_*, corresponds to the inequality only between those individuals that are members of group *i* and is given by:

$T_{i}=\sum_{p=1}^{n_{i}} r_{ip}$, （11）

with:

$r_{ip}=\frac{y_{ip}}{Y_{i}}\log\left[\left(\frac{y_{ip}}{Y}\right)/\left(\frac{1}{n_{i}}\right)\right]$ （12）

Therefore, this study measures the intra group, inter group and overall differences of China's basic medical and health service level based on the Thiel index. The specific calculation formula is as follows:

$T=\sum_{i=1}^{n} \frac{F_{i}}{F}\sum_{j=1}^{n_{i}} \left.\left\{\frac{F_{ij}}{F_{i}}\times\ln\left[\left(\frac{F_{ij}}{F_{i}}\right)/\left(\frac{P_{ij}}{P_{i}}\right)\right]\right.\right\}\text{+}\sum_{i=1}^{n} \frac{F_{i}}{F}\ln\left[\left(\frac{F_{i}}{F}\right)/\left(\frac{P_{i}}{P}\right)\right]=T^{W}+T^{B}$ （13）

Among them, *T* represents the overall difference of basic Chinese medical and health service level, *F* represents the basic medical and health service level, *F_i_* represents the basic medical and health service level in group *i*, and *F_ij_* represents the basic medical and health service level in group *i*, province and region *j*. *n* refers to regional grouping, and *n_i_* refers to *n_i_* provinces in group *i*. *P* represents the population, *P_i_* represents the population of the region of group *i*, and *P_ij_* represents the population of the province of group *j*.

In order to further study the contribution of interval differences and intra interval differences to the overall difference, the inter regional contribution rate is defined as the ratio of inter regional Theil index to the overall Theil index, namely $\frac{T^{B}}{T}$; The contribution rate within the region is the ratio of the Thiel index within the region to the overall Thiel index, namely $\frac{T^{W}}{T}$. In addition, the contribution rate of each sub region in the region is defined as the ratio of the weighted Thiel index of each sub region to the total Thiel, namely $\left(\frac{F_{j}}{F}\right)\times\left(\frac{T^{wi}}{T}\right)$.

(3) Convergence analysis

The formula for calculation is as follows:

（14）

$CV=\frac{S}{F{}_{it}}$ （15）

Where, *i* represents the different provinces or regions, *N* represents the number of provinces or regions, *S* represents the standard deviation,$F_{it}$ represents the score of the basic medical and health service level of region *i* at time *t*, $\mathop{F_{it}}\limits^{\text{—}}$ represents the average score of the basic medical and health service levels.

*σ*-convergence involves the process of diminishing disparities over time among samples, indicating that differences between economies are decreasing and tending towards a state of equilibrium. *β*-convergence, originating from the theory of economic convergence in neoclassical economics, was initially used to study the uniformity of economic growth among regions or countries (48). Furthermore, *β*-convergence is subdivided into absolute *β*-convergence and conditional *β*-convergence: absolute *β*-convergence suggests that economies with similar structures will converge over time to the same growth path and steady state; conditional *β*-convergence takes into account differences in economic structures, positing that different economies will tend towards their specific steady-state levels influenced by external conditions (47).

In this study, the convergence of the level of basic medical and health services in China was investigated by *β* convergence form. *β* convergence is derived from the theory of economic convergence in neoclassical economy and was used in the early discussion of economic growth convergence between regions or countries (47-49). For the level of medical and health services between provinces, if there is convergence, it shows that the speed of the improvement of the level of medical and health services in a province is negatively related to the initial level, which makes the difference in the level of medical and health services between the two provinces narrowing. Due to different application prerequisites, *β* Convergence is divided into absolutely *β* Convergence and conditional *β* Convergence. In dealing with short-term panel data, where the time span is *T*=8, and considering the high similarity in the economic structures of the various provinces and regions of China, this study opts to test for the presence of absolute *β*-convergence. The hypothesis of absolute *β*-convergence is grounded in the neoclassical growth model, which predicts that all economies, regardless of their starting points, will ultimately converge to the same equilibrium growth path, assuming that all regions will reach a common steady-state growth rate in the long term. In this context, absolute *β*-convergence is typically verified by examining the negative correlation between economic growth rates and initial levels. If the poorer provinces (with lower initial economic levels) grow faster, this would support the notion of absolute *β*-convergence. In this study, the presence of absolute convergence would indicate that the basic medical and health service levels across the provinces are narrowing their initial disparities and converging towards a common level. This convergence is usually tested through the *β* coefficient in regression analysis; if this coefficient is significantly negative, it would signify the presence of absolute *β*-convergence.

In this study, the absolutely *β* convergence test is conducted, and the formula is as follows:

$\ln\left(\frac{F_{i,t+1}}{F_{it}}\right)=\alpha+\beta\ln F_{it}+\mu_{i}+\eta_{t}+\varepsilon_{it}$ （16）

Where, *i*=1,2,3,..., *N* represents the *i* region; *t*=1,2,3,..., *T* represents time, $\ln\left(\frac{F_{i,t+1}}{F_{it}}\right)$ represents the annual growth rate of the medical and health service level score of the *i* region in the *t*~*t*+1 period. $F_{i,t+1}$ represents the score of medical and health service level of region *i* in period *t*+1, and $F_{it}$ represents the score of basic medical and health service level of region *i* in period *t*. *α* Is a constant term, *β* Is the coefficient to be estimated, $\mu_{i}$ is the regional effect, $\eta_{t}$ is the time effect, and $\varepsilon_{it}$ is the random disturbance term. if *β*< 0, and the significance test shows that there is obviously convergence, and the convergence rate is:

$v=-\frac{\ln(1+\beta)}{T}$ （17）
